# Genome Constellations Recovered Following In Vitro Co-Infection and Serial Passage of Bluetongue Virus Serotypes 1 and 16

**DOI:** 10.3390/v18091029

**Published:** 2026-09-16

**Authors:** Gloria Plebani, Anna Salvaggiulo, Federica Pizzurro, Liana Teodori, Alessandra Leone, Lorenza Brandimarte, Mariassunta Iannetta, Simone Pulsoni, Giacomo Vecchiato, Claudia Casaccia, Alessio Lorusso

**Affiliations:** 1 Department of Bioscience and Agro-Food and Environmental Technology, University of Teramo, 64100 Teramo, Italy; g.plebani@izs.it (G.P.); a.salvaggiulo@izs.it (A.S.); 2 Istituto Zooprofilattico Sperimentale dell’Abruzzo e del Molise, 64100 Teramo, Italy; f.pizzurro@izs.it (F.P.); l.teodori@izs.it (L.T.); a.leone@izs.it (A.L.); l.brandimarte@izs.it (L.B.); m.iannetta@izs.it (M.I.); s.pulsoni@izs.it (S.P.); c.casaccia@izs.it (C.C.); 3 Estilos s.r.l., 30172 Venice, Italy; giacvecchiato@gmail.com

**Keywords:** bluetongue virus, reassortment, *Orbivirus*, co-infection, genome constellation, serial passage

## Abstract

Bluetongue virus (BTV) is a segmented RNA virus capable of genome reassortment during co-infection. We characterized genome constellations recovered after co-infection of Vero cells with western BTV-1 and eastern BTV-16 strains followed by three serial passages. Of 157 plaques, 46 showed simultaneous detection of BTV-1 and BTV-16 Seg-2 and were excluded because a single parental background could not be established. Among 111 Seg-2-monotypic plaques, 20 were identified as reassortants, representing 18.0% of the evaluable plaques and 12.7% of all plaques collected. Across the 20 recovered reassortants, 111 of 200 segment assignments derived from BTV-1 and 89 from BTV-16. BTV-1-derived Seg-7 was observed in 18 of 20 plaques and BTV-16-derived Seg-3 in 14 of 20 plaques; 18 distinct genome constellations were observed. Because all plaques originated from a single co-infection and were sampled after three serial passages without parallel single-virus passage controls, the observed segment distributions integrate reassortment and post-reassortment selection and cannot separate these processes. These data demonstrate recovery of diverse BTV-1/BTV-16 reassortant genomes under the experimental conditions but do not establish the true reassortment frequency or reproducible segment-association patterns.

## 1. Introduction

Bluetongue virus (BTV), the causative agent of bluetongue (BT), belongs to the genus *Orbivirus* within the family *Sedoreoviridae* [1,2]. BT is a non-contagious arboviral disease affecting domestic and wild ruminants and is transmitted by competent species of biting midges (*Culicoides* spp.). Consequently, the global distribution of BTV is closely associated with that of its insect vectors [1,3]. The BTV genome is composed of 10 double-stranded (ds) RNA segments (Seg-1 to Seg-10), enclosed within three concentric protein layers forming the subcore, core, and outer capsid of the virion. Variability in the outer capsid proteins, primarily VP2 and to a lesser extent VP5, determines viral serotype specificity. To date, 36 BTV serotypes have been described, including 24 classical and 12 atypical serotypes [4,5]. In addition, phylogenetic analyses of the VP2 gene have identified two major geographic lineages within individual serotypes, referred to as eastern (e) and western (w) topotypes, broadly associated with viruses originating from Australia and the Middle/Far East or from Africa and the Americas, respectively [6].

Since 1998, southern Europe has experienced multiple incursions of distinct BTV serotypes, strains, and topotypes. These have spread mainly through two routes: eastern topotypes (BTV-1e, BTV-9e, BTV-16e) introduced through Turkey into Greece and Bulgaria, and western topotypes (BTV-1w, BTV-2w, BTV-4w) likely introduced through wind-borne dissemination of infected Culicoides from northern Africa [7]. In addition, vaccine-derived strains originating from modified live-attenuated vaccines (MLVs) have circulated simultaneously with wild-type strains in Europe [8,9,10].

The segmented nature of the BTV genome enables reassortment when a host cell is co-infected by two or more distinct viral strains or serotypes. The co-circulation of multiple BTV strains in animal hosts and vector populations therefore creates opportunities for genome segment exchange, potentially generating viruses with altered biological, antigenic, or epidemiological characteristics [9,11,12,13].

Reassortment has been documented in both arthropod [14,15,16] and mammalian hosts [17,18], as well as under experimental in vitro conditions [12,19,20]. Although reassortment between serologically unrelated BTV strains appears relatively flexible and can theoretically involve all genome segments [12], reassortment between genetically distant strains belonging to different topotypes may be subject to additional constraints, as suggested by reverse genetics studies [21]. Nevertheless, reassortant viruses involving eastern and western topotypes have been identified in the field [9,22,23].

Studies on segmented viruses have shown that the composition of reassortant genomes can be shaped by multiple processes, including constraints acting during genome packaging as well as selection after reassortment [24,25,26,27]. Consequently, segment distributions recovered after experimental passage need not reflect the probability of segment assortment alone.

Considering these observations, we performed an in vitro co-infection assay using two BTV serotypes of distinct geographic origin, eastern BTV-16 and western BTV-1, to characterize the reassortant genome constellations recovered after serial passage in Vero cells. The BTV-1w strain used in this study was first detected in Sardinia (Italy) in 2006 and has been reported to remain genetically and antigenically stable over time [28,29].

In contrast, BTV-16e was first identified in the Apulia region of southeastern Italy in 2002 and was hypothesized to derive from reassortment events involving vaccine strains BTV-16e and BTV-2w, both of which had been used for several years in multivalent vaccination campaigns in Israel [9,30]. Subsequently, outbreaks caused by BTV-16 in Sardinia and mainland Italy during 2004 were associated with a strain genetically indistinguishable from the BTV-16 vaccine strain used during vaccination campaigns in Italy. Notably, this vaccine-derived strain was associated with severe disease in vaccinated animals and produced a viremia of sufficient magnitude and duration to allow infection of feeding *Culicoides* vectors, thereby facilitating its spread in the field [31,32]. Following these observations, the use of the BTV-16 vaccine strain was discontinued.

## 2. Materials and Methods

### 2.1. Cells and Viruses

KC cells [33], derived from embryos of colonized Culicoides sonorensis [34], were maintained in modified Schneider’s Drosophila medium supplemented with 15% fetal bovine serum (FBS), 100 IU/mL penicillin, and 100 μg/mL streptomycin, and incubated at 27 °C. Vero cells (ATCC CCL-81; American Type Culture Collection, Manassas, VA, USA) were maintained in Dulbecco’s Modified Eagle Medium (DMEM) supplemented with 5% FBS and 25 μg/mL penicillin/streptomycin (P/S), and incubated at 37 °C in a humidified atmosphere containing 5% CO_2_. The viral strains used in this study included a western topotype BTV-1 strain isolated in the province of Viterbo, Italy, in 2013 (BTV-1 LAZ2013; NCBI accession numbers KJ577124–KJ577133) and an eastern topotype BTV-16 strain isolated in the province of Macerata, Italy, in 2007 (BTV-16 85719/2007; NCBI accession numbers MH999872–MH999881). Virus stocks were generated by a single passage on confluent KC cell monolayers for 10 days at 27 °C, followed by two passages on confluent Vero cell monolayers at 37 °C in a humidified atmosphere containing 5% CO_2_. Prior to co-infection, infectious titres of the two parental virus stocks were determined to define the inocula used for the experiment.

### 2.2. In Vitro Co-Infection Assay

Vero cells were seeded in 12-well plates and co-infected with BTV-1w and BTV-16e at a multiplicity of infection (MOI) of 1 for 2 h. An MOI of 1 was selected to promote co-infection while maintaining balanced representation of both parental viruses. Following adsorption, the inocula were removed and replaced with 1 mL of fresh growth medium. At 24 h post-infection (PI), when a clear cytopathic effect (CPE) was observed, supernatant and cellular debris were collected and pooled (hereafter referred to as the ‘reassortant mix’). A 5 µL aliquot of the reassortant mix was subsequently passaged three consecutive times in Vero cells. At each passage, 5 µL of the previous culture supernatant was used as inoculum in 12-well plates containing confluent Vero cell monolayers, and supernatant together with cellular debris was harvested at 48 h post-infection. Following the third passage, the supernatant was collected and filtered through a 0.2 µm membrane filter to reduce the presence of aggregated particles and cellular debris. The filtered suspension was then used to infect Vero cells seeded in 6-well plates, followed by agar overlay as previously described [12]. Individual well-isolated plaques were picked through the agar overlay, resuspended in 350 µL of DMEM, and stored at 4 °C until further analysis. All plaques that were sufficiently well isolated to permit individual picking through the agar overlay were collected; no additional selection based on plaque size, morphology, or position was applied.

### 2.3. Detection of Reassortant Viruses In Vitro

Viral RNA was extracted from 140 µL of each plaque resuspension using the QIAamp Viral RNA Mini Kit (Qiagen^TM^, Hilden, Germany), according to the manufacturer’s instructions. A total of 157 plaques were collected and analyzed. RNA extracted from each individual plaque was initially screened using a real-time RT-qPCR assay detecting all BTV serotypes [35], followed by typing with the VetMAX™ European BTV Typing Kit (Applied Biosystems, Austin, TX, USA), which detects Seg-2 of serotypes 1, 2, 4, 6, 8, 9, 11 and 16 circulating or previously circulating in the Mediterranean basin. Plaques positive for both BTV-1 and BTV-16 Seg-2 were excluded from further analysis to reduce the likelihood of analyzing clearly mixed viral populations. Because a monotypic Seg-2 result does not by itself demonstrate clonality across the complete genome, the remaining nine segments were characterized individually. Segment-specific RT-PCR assays were performed using the primer sets listed in Table 1. The primer pairs were designed to amplify regions shared by the two parental strains, permitting amplification of both BTV-1 and BTV-16 templates for each segment other than Seg-2. Prior to analysis of plaque samples, all primer sets underwent validation using known positive controls derived from BTV-1 LAZ2013 and BTV-16 85719/2007 infected cells. Specificity was confirmed by testing each primer pair against a single-serotype template to exclude cross-reactivity. The amplified regions exhibited nucleotide identity levels ranging from approximately 94–95% (Seg-1, Seg-3) to >96% (Seg-7) between serotypes, providing sufficient sequence divergence for unambiguous parentage determination. The resulting amplicons were cloned into the pGEM-T Easy vector (Promega, Madison, WI, USA) and sequenced in both directions using internal primers. Sequence reads were assembled and analyzed using the DNASTAR Lasergene version 14 (DNASTAR Inc., Madison, WI, USA), and parental origin was assigned by comparison with the corresponding parental BTV-1 and BTV-16 sequences. Parental origin assignment was determined by pairwise sequence comparison of each amplicon against the corresponding genomic segments of both reference strains. Segments were assigned to the parental virus displaying the highest sequence identity in the amplified region; no fixed percentage-identity cutoff was applied. Only segments for which comparison with both parental references allowed unambiguous assignment were used to define complete genome constellations. Ten bacterial colonies per cloning plate were sequenced as an internal verification step. Clone-level concordance was not treated as a separate quantitative endpoint; therefore, no retrospective frequency of minor discordant clone sequences is reported.

### 2.4. Data Analysis and Interpretation

All plaques analyzed in this study originated from a single initial co-infection well and were therefore treated as subsamples from one experiment rather than as independent biological replicates. Data were summarized descriptively using counts and percentages. Because the experiment contained only one biological cluster (the single co-infection well), no between-experiment variance could be estimated and no inferential, hierarchical, cluster-corrected, or correlation analyses were performed. For the same reason, no formal statistical comparison was made between the proportions of reassortant plaques recovered on BTV-1- and BTV-16-derived Seg-2 backgrounds. Segment-origin counts were derived from the segment-origin assignments of the 20 recovered reassortant plaques.

## 3. Results

### 3.1. Recovery of Reassortant Plaques After Serial Passage

A total of 46 of 157 plaques (29.3%) showed simultaneous detection of BTV-1 and BTV-16 Seg-2 and were excluded because they could not be assigned to a single Seg-2 parental background. Among the remaining plaques, 34 of 157 (21.7%) were monotypic for BTV-1 Seg-2 and 77 of 157 (49.0%) were monotypic for BTV-16 Seg-2. These 111 Seg-2-monotypic plaques were selected for characterization of the remaining nine genome segments.

**Figure 1:**
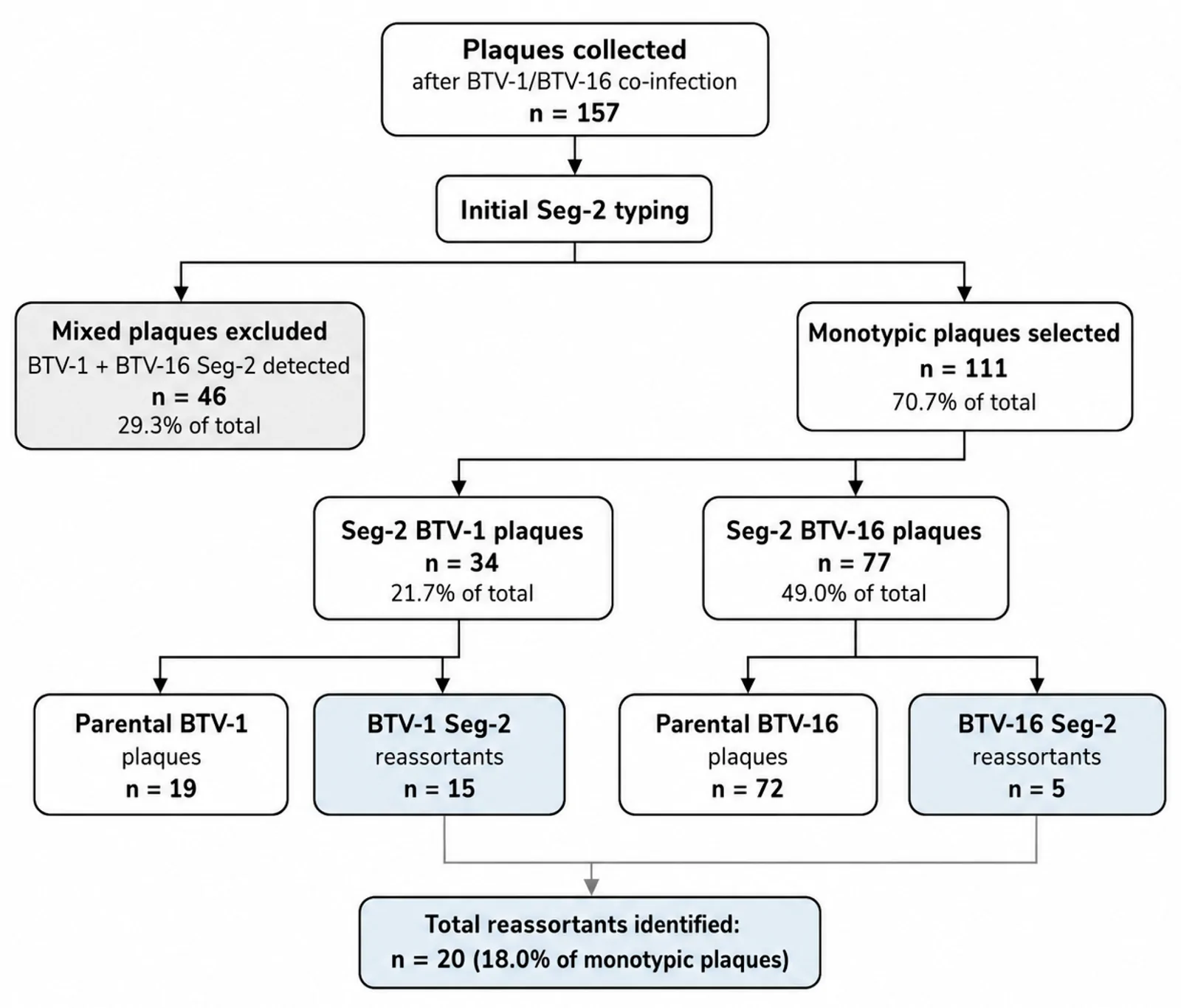
Workflow and outcome of plaque screening after in vitro co-infection with BTV-1 and BTV-16 followed by serial passage. A total of 157 plaques were collected and screened by Seg-2 typing. Forty-six plaques showed simultaneous detection of BTV-1 and BTV-16 Seg-2 and were excluded because their Seg-2 background could not be assigned unambiguously. The remaining 111 Seg-2-monotypic plaques were further characterized, leading to the definitive identification of 20 reassortant plaques: 15 carrying BTV-1-derived Seg-2 and five carrying BTV-16-derived Seg-2. These 20 plaques represent 18.0% of the Seg-2-monotypic plaques evaluated and 12.7% of all plaques collected; the true reassortment frequency remains unknown because the 46 mixed plaques were not resolved.

Among the 111 Seg-2-monotypic plaques evaluated, 20 were definitively identified as reassortants, corresponding to 18.0% of the Seg-2-monotypic plaques and 12.7% of all 157 plaques collected. Because the 46 plaques with mixed Seg-2 detection were excluded and may themselves have contained reassortant viruses, the true reassortment frequency cannot be determined from this dataset. Of the 34 plaques carrying BTV-1-derived Seg-2, 15 (44.1%) were reassortants, whereas 5 of 77 (6.5%) plaques carrying BTV-16-derived Seg-2 were reassortants. These proportions describe plaque recovery after the complete co-infection and serial-passage procedure and should not be interpreted as independent probabilities of reassortment. The plaque-screening workflow and outcome are summarized in Figure 1, and a summary of plaque analysis is provided in Table S1.

### 3.2. Segment Origins and Recovered Genome Constellations

Across the 20 recovered reassortant plaques, 111 of 200 segment assignments (55.5%) were derived from BTV-1 and 89 of 200 (44.5%) from BTV-16. Because the ten segments are linked within each genome constellation, this aggregate distribution is presented descriptively and is not interpreted as a statistical test of parental dominance. At the individual-segment level, BTV-1-derived Seg-7 was observed in 18 of 20 plaques (90.0%), whereas BTV-16-derived Seg-3 was observed in 14 of 20 plaques (70.0%) (Table 2). These values are descriptive counts of the sampled plaques and are not interpreted as evidence of preferential assortment, segment compatibility, or differential fitness.

The genome constellations recovered among the 20 reassortant plaques are reported in Table 3. Eighteen distinct constellations were observed. Two constellations (C1 and C15) were each recovered from two plaques. Because all plaques derived from the same initial co-infection and subsequent serial passages, recovery of the same constellation from more than one plaque cannot be interpreted as evidence that the same reassortant genome arose independently more than once; clonal expansion of an ancestral reassortant followed by repeated plaque sampling is an alternative explanation. The recovered genome constellations are also provided in Table S2.

### 3.3. Stratification of Recovered Reassortants by Seg-2 Origin

After stratification by Seg-2 origin, among the 15 plaques carrying BTV-1-derived Seg-2, BTV-1-derived Seg-6, Seg-7, and Seg-8 were observed in 12/15, 13/15, and 11/15 plaques, respectively. All five plaques carrying BTV-16-derived Seg-2 contained BTV-16-derived Seg-1, Seg-3, Seg-4, Seg-6, and Seg-8 together with BTV-1-derived Seg-7 (Table 4). These counts are descriptive only and do not establish segment preference or compatibility.

## 4. Discussion

This study used field isolates belonging to western and eastern BTV topotypes to characterize reassortant genome constellations recovered after in vitro co-infection and three serial passages in Vero cells. Previous experimental studies have shown that BTV reassortment can generate diverse genome constellations and that segment distributions recovered after co-infection are not always uniform [12,19,20]. The present experiment likewise recovered multiple BTV-1/BTV-16 reassortant genomes. However, the design was not intended, and does not permit estimation of a population-level reassortment frequency or identification of reproducible segment-association patterns.

The main limitation is that all 157 plaques originated from a single co-infection well. The plaques are therefore subsamples from one experimental population rather than independent biological observations. This distinction affects interpretation of the 20 reassortant plaques, the segment percentages, and the repeated genome constellations. In particular, recovery of C1 or C15 from two plaques does not demonstrate that the same constellation arose independently twice; a single ancestral reassortant may have expanded during passage and subsequently been sampled more than once. Independent co-infection replicates would be required to determine whether particular constellations or segment combinations are reproducibly recovered.

The experimental workflow included three serial passages in Vero cells, each initiated with 5 µL of the preceding 1 mL culture harvest (0.5% of the harvested volume). This imposed a substantial sampling bottleneck that could reduce rare genotypes and amplify differences in replication fitness. Because genotype-specific frequencies and infectious titres were not measured at each passage, the magnitude of genotype loss cannot be quantified retrospectively. In addition, no parallel serial-passage controls of BTV-1 and BTV-16 alone were included. Matched growth-kinetic data for the exact parental stocks under the Vero-cell conditions used for serial passage were also not available, so differential parental fitness during passage cannot be quantified from this dataset. Consequently, the segment distributions observed at plaque isolation integrate at least two processes that this design cannot separate: generation and packaging of reassortant genomes during co-infection and subsequent selection during serial passage in Vero cells. The data are therefore reported as segment compositions recovered after the complete co-infection/passage workflow rather than as evidence of reassortment bias or segment compatibility.

Among the 111 Seg-2-monotypic plaques that could be evaluated further, 15 of 34 plaques with a BTV-1 Seg-2 background were reassortants compared with 5 of 77 plaques with a BTV-16 Seg-2 background. This difference in observed proportions is a feature of the recovered plaque population. Because all plaques are subsamples of the same experimental population rather than independent biological replicates, no chi-square or other inferential test was performed. The difference therefore cannot be interpreted as evidence that reassortment intrinsically occurs more readily on a BTV-1 Seg-2 background; it may instead reflect differential replication or survival during passage, plaque-sampling effects, or a combination of these factors.

Across the 20 recovered reassortants, 111 of 200 segment assignments were derived from BTV-1 and 89 from BTV-16. This aggregate count is descriptive only because the ten segments are linked within each plaque. BTV-1-derived Seg-7 was present in 18/20 plaques and BTV-16-derived Seg-3 in 14/20 plaques; these observations do not establish that either segment was preferentially assorted or selected.

Comparison with previous BTV studies further emphasizes the distinction between recovered genome constellations and mechanisms of reassortment. Nomikou et al. [13], analyzing more than 150 field isolates from Europe, documented widespread reassortment under natural epidemiological and evolutionary pressures and identified non-random associations among some segment pairs. In an experimental setting, Kopanke et al. [20] reported shifts in segment frequencies and preferred segment combinations after in vitro co-infection of endemic BTV strains. The present study differs from both because a single BTV-1w/BTV-16e co-infection was followed by three Vero-cell passages before plaque isolation. A directly testable hypothesis arising from the present dataset is that the final segment compositions reflect a combination of initial genome assortment and subsequent cell-culture selection; replicated co-infections with matched parental controls and sampling before and after each passage would allow these components to be separated.

The present data should therefore be interpreted as a descriptive snapshot of the reassortant population recovered under a specific in vitro workflow. The recovery of 18 distinct constellations among 20 reassortant plaques documents genomic diversity in the sampled population, but the experiment cannot determine how many independent reassortment events generated those plaques or whether the same segment combinations would recur in replicate experiments. Likewise, because 46 of 157 plaques showed mixed Seg-2 detection and were excluded, 20/111 (18.0%) represents the proportion of definitively identified reassortants among evaluable Seg-2-monotypic plaques, while 20/157 (12.7%) is the proportion definitively identified among all collected plaques; neither value should be interpreted as the true reassortment frequency.

Future studies should include independent co-infection replicates, parallel serial-passage controls for each parental virus, and matched growth-kinetic experiments using the exact parental stocks in Vero cells. Sampling the viral population before and after each passage would directly test whether specific constellations are generated during reassortment or enriched during subsequent culture. Experiments in both mammalian and Culicoides-derived cells, particularly with strains that co-circulate in the field, would then test whether any segment compositions are reproducibly recovered across biologically relevant environments.

Within these limitations, the study shows that co-infection of Vero cells with BTV-1w and BTV-16e can yield a diverse set of reassortant genomes that remain recoverable after serial passage. The principal value of the dataset lies in documenting these recovered genome constellations and in identifying hypotheses that can be tested in replicated experiments, rather than in demonstrating non-random reassortment or fixed segment-compatibility rules.

## Figures and Tables

**Table 1:** Primers used for amplification and sequencing of BTV genome segments other than Seg-2.

Segment	Primer Name	Sequence	Amplicon Size (bp)
1	BTVS1_321-338f	CCGTGGAGTGAACTTGAT	638
	BTVS1_942-959r	ATAAAACGATGACCGAAA	
	BTVS1_321-338f	CCGTGGAGTGAACTTGAT	218
	feeding-1r	ATTTACGAAGTCAGCCATAAC	
3	BTVS3_187-206f	TTGACTTCACAGTGCCAGAT	735
	BTVS3_902-922r	TATTCTCCTGTAGGTAAACAC	
	BTVS3_187-206f	TTGACTTCACAGTGCCAGAT	321
	feeding-3r	AACACTTCTGGTTCCGCAAC	
4	BTVS4_28-47	GGATATGAAAGTTTAAACGA	579
	BTVS4_588-607	TCACCGCATCCAACATAGTG	
	feeding-4f	ATCCAGCGTGGGCGATGAA	248
	feeding-4r	TCATCTTAAACCACCAGAACA	
5	BTVS5_491-510f	CAAATCGTGAATCCAACATT	448
	BTVS5_921-939r	TCATCACCCGATCTCCTCA	
	feeding-5f	TTTTGGCTATTTCACCACAATGG	289
	feeding-5r	CATCCCACTTTTGCGGTAATC	
6	BTVS6_224-243f	GGCGAATCTGTGAAACAAGC	589
	BTVS6_795-813r	ATCACCTTTTTGAGTTTAT	
	feeding-6f	GATGTGTTCTTCTTTCATTGT	262
	feeding-6r	CCTAAATAGATTGGATGTA	
7	BTVS7_328-346	AGGCGGCGAATGAGATTGC	763
	BTVS7_1071-1091	TAACCCACACCCGTGCAAAGT	
	BTVS7_328-346	AGGCGGCGAATGAGATTGC	267
	feeding-7r	TTTTCAATCCTTCTCCAAAC	
8	BTVS8_569-588f	TGGATGGATGATGATGAAGC	470
	BTVS8_1020-1039r	AAAAGCAACACGCTCACACG	
	feeding-8f	AAGGATGAACGGTTTATGAG	173
	BTVS8_1020-1039r	AAAAGCAACACGCTCACACG	
9	BTVS9_448-467f	GAATCTAAATACGGTACGAA	572
	BTVS9_1001-1020r	CTGGACCCTTTAGAGGTGAT	
	feeding-9f	AGTTGATTGGACAGAAAGTGA	213
	feeding-9r	GCGTTCTCCAGGTCCTTTTG	
10	BTVS10_268-287f	AGCGTTTCGTGATGATGTGA	461
	BTVS10_712-729r	CGCCACTCTACCTACTGA	

**Table 2:** Parental origin of segment assignments among the 20 recovered BTV reassortant plaques.

Seg.	BTV-1 Origin, *n* (%)	BTV-16 Origin, *n* (%)
Seg-1	9 (45.0)	11 (55.0)
Seg-2	15 (75.0)	5 (25.0)
Seg-3	6 (30.0)	14 (70.0)
Seg-4	8 (40.0)	12 (60.0)
Seg-5	12 (60.0)	8 (40.0)
Seg-6	12 (60.0)	8 (40.0)
Seg-7	18 (90.0)	2 (10.0)
Seg-8	11 (55.0)	9 (45.0)
Seg-9	9 (45.0)	11 (55.0)
Seg-10	11 (55.0)	9 (45.0)

Counts and percentages are descriptive summaries of the 20 reassortant plaques recovered from a single co-infection experiment. The 200 segment assignments are nested within 20 genome constellations; neither plaques nor segment assignments represent independent biological observations.

**Table 3:** Genome constellations recovered among BTV reassortant plaques.

Constellation ID	Seg-1	Seg-2	Seg-3	Seg-4	Seg-5	Seg-6	Seg-7	Seg-8	Seg-9	Seg-10	*n*
C1	1	1	1	1	16	1	1	1	1	16	2
C2	16	1	16	1	1	1	1	1	1	1	1
C3	16	1	16	1	16	1	16	1	16	1	1
C4	16	1	16	1	1	16	1	16	16	16	1
C5	16	1	16	16	1	1	1	1	16	1	1
C6	16	1	16	16	16	1	1	16	1	1	1
C7	16	1	16	16	1	1	1	1	16	16	1
C8	1	1	16	1	1	1	1	1	1	1	1
C9	1	1	16	16	1	1	1	1	1	1	1
C10	1	1	16	1	1	1	1	1	16	1	1
C11	1	1	1	16	1	16	1	1	1	16	1
C12	1	1	1	16	16	1	1	1	16	1	1
C13	1	1	1	16	1	16	1	16	1	16	1
C14	1	1	1	1	1	1	16	16	16	1	1
C15	16	16	16	16	16	16	1	16	16	16	2
C16	16	16	16	16	16	16	1	16	16	1	1
C17	16	16	16	16	1	16	1	16	16	1	1
C18	16	16	16	16	1	16	1	16	1	16	1

Segment origin is indicated as 1 for BTV-1 and 16 for BTV-16. The final column (*n*) indicates the number of sampled plaques with each genome constellation and should not be interpreted as the number of independent reassortment events.

**Table 4:** Segment assignments among recovered reassortant plaques after stratification by Seg-2 origin.

Segment	BTV-1 Seg-2 Reassortants, *n* = 15: BTV-1 Origin *n* (%)	BTV-1 Seg-2 Reassortants, *n* = 15: BTV-16 Origin *n* (%)	BTV-16 Seg-2 Reassortants, *n* = 5: BTV-1 Origin *n* (%)	BTV-16 Seg-2 Reassortants, *n* = 5: BTV-16 Origin *n* (%)
Seg-1	9 (60.0)	6 (40.0)	0 (0.0)	5 (100.0)
Seg-2	15 (100.0)	0 (0.0)	0 (0.0)	5 (100.0)
Seg-3	6 (40.0)	9 (60.0)	0 (0.0)	5 (100.0)
Seg-4	8 (53.3)	7 (46.7)	0 (0.0)	5 (100.0)
Seg-5	10 (66.7)	5 (33.3)	2 (40.0)	3 (60.0)
Seg-6	12 (80.0)	3 (20.0)	0 (0.0)	5 (100.0)
Seg-7	13 (86.7)	2 (13.3)	5 (100.0)	0 (0.0)
Seg-8	11 (73.3)	4 (26.7)	0 (0.0)	5 (100.0)
Seg-9	8 (53.3)	7 (46.7)	1 (20.0)	4 (80.0)
Seg-10	9 (60.0)	6 (40.0)	2 (40.0)	3 (60.0)

Counts and percentages are shown separately for reassortant plaques carrying BTV-1-derived Seg-2 (*n* = 15) and BTV-16-derived Seg-2 (*n* = 5). They are descriptive only and do not represent independent biological replicates.

## Data Availability

Sequence data generated for segment assignment during this study are available from the corresponding author upon reasonable request. Reference genome sequences of the parental BTV-1 and BTV-16 strains are available in GenBank under accession numbers KJ577124–KJ577133 and MH999872–MH999881, respectively.

## Supplementary Materials

The following supporting information can be downloaded at https://www.mdpi.com/article/10.3390/v18091029/s1. Table S1: Summary of plaque analysis; Table S2: Reassortant genome constellations recovered after in vitro co-infection and serial passage.
